# Transcriptome Analysis and Its Application in Screening Genes Related to the Growth and Development of *Sarcomyxa edulis*

**DOI:** 10.3390/jof11100750

**Published:** 2025-10-20

**Authors:** Wanzhu Jiang, Xiao Huang, Peng Wang, Bilal Ahmad, Ting Yang, Ziyuan Wang, Tianyu Ren, Jize Xu

**Affiliations:** 1 Agricultural College, Jilin Agricultural Science and Technology College, Jilin 132101, China; jiangwanzhu@jlnku.edu.cn (W.J.);; 2 Economic Plants Institute, Jilin Academy of Agricultural Sciences (Northeast Agricultural Research Center of China), Changchun 130033, China; huangxiaoxiwang@sina.com (X.H.);; 3 College of Plant Protection, Jilin Agricultural University, Changchun 130118, China

**Keywords:** *Sarcomyxa edulis*, transcriptome sequencing, growth and development, tryptophan metabolism

## Abstract

*Sarcomyxa edulis* is a characteristic edible and medicinal mushroom found in Northeast China that is highly valued by consumers for its tender texture, pleasant flavor, and high nutritional value. To gain a deeper understanding of the molecular mechanisms underlying the development of *S. edulis* fruiting bodies, this study utilized the Illumina NovaSeq platform to perform transcriptome sequencing at three growth and development stages of *S. edulis* strain SE8, namely primordia (SE8–P), fruiting body differentiation (SE8–F), and mature fruiting body (SE8–M). A total of 54.67 Gb of clean data was obtained, with a GC content of around 51%. After assembly, 36,423 Unigenes were obtained. Functional annotation was performed on the Unigenes, resulting in 21,206 Unigene annotation results. Differential expression gene analysis showed that 79,606 and 523 DEGs were annotated in at least one database during the SE8–P vs. SE8–F, SE8–F vs. SE8–M, and SE8–P vs. SE8–M processes, respectively. Among these, the genes encoding aldehyde dehydrogenase and fungal hydrophobins were consistently downregulated, playing a negative regulatory role in the growth and development of *S. edulis*. The genes encoding glycoside hydrolase and AB hydrolase superfamily proteins were consistently upregulated, playing a positive regulatory role in growth and development. Among these, the genes encoding aldehyde dehydrogenase were annotated to the Tryptophan metabolism (ko00380) pathway through KEGG, suggesting that aldehyde dehydrogenase regulates indoacetate formation in the fruiting body of *S. edulis*. The accuracy of RNA–Seq and DEG analysis was validated using quantitative PCR. This study enriches our knowledge of the genetic information and provides a theoretical basis for the molecular mechanisms of fruiting body development of *S. edulis*.

## 1. Introduction

*Sarcomyxa edulis* are widely distributed in the Northern Hemisphere, especially in the forests of Northeast China, where they are most abundant [1]. Their fruiting bodies are fleshy and delicious, with extremely high nutritional and medicinal value [2,3,4]. Currently, a lot of work has been carried out in the breeding of excellent *S. edulis* strains [5,6,7,8] and genetic mechanism research [9,10,11].

The fruiting body is the main edible part of *S. edulis* and an important structure for growth and development. Its developmental regulation mechanisms have important research value. Currently, there is relatively little research on the formation mechanisms of fruiting bodies in *S. edulis*, and only Duan [12] has conducted a systematic analysis of different growth and development stages. The development of fruiting bodies is an extremely complex process, which is regulated not only by environmental factors but also by genetic factors [13]. Although growth and development mechanisms have been studied in various fungi, their molecular mechanisms are still not fully understood. However, through the unremitting efforts of researchers in various countries, a large number of functional genes related to fruiting body development have been discovered and identified [14]. Pelkmans [15] found in a study on *Schizophyllum commune* that *Bri1* and *Hom1* play a role in the later stages of development, while *Wc–2*, *Hom2*, and *Fst4* play a role in the early stages of development. Ohm [16] also found that, in *S. commune*, seven genes, including *hom1*, *hom2*, *c2h2*, *gat1*, *bri1*, *fst3*, and *fst4*, play a role in the development of primordia and fruiting bodies. Boulianne [17] found that *cgl1* and *cgl2* genes regulate the formation of fruiting bodies during the development of *Coprinus cinereus*. *cgl2* was expressed during hyphal formation, while *cgl1* was expressed during primordia and subsequent development. Wagemaker [18] found that hydrophobic protein genes, urease genes, and mannitol dehydrogenase genes were involved in the formation process of *Agaricus bisporus* fruiting bodies at different developmental stages. Meanwhile, Wu [19] discovered various amino acid synthases, transcription factors, GTP-binding proteins, etc., in *A. bisporus*, which were key genes related to fruiting body development. In addition, Almási [20] and Krizsán [21] found that heat shock proteins, small secreted proteins, kinases, F-Box proteins, and genes related to cell wall remodeling may be involved in the development of fruiting bodies in other basidiomycetes. Pei [22] discovered and identified a gene, g13394, in *Pleurotus ostreatus* that can promote hyphal growth and accelerate fruiting body development.

*S. edulis*, as a characteristic low-temperature edible and medicinal mushroom found in Northeast China, has great development prospects. Our team has been engaged in genetic breeding and cultivation research. We have collected and evaluated the germplasm resources of *S. edulis*, optimized cultivation formulas and techniques, analyzed the biological characteristics of core germplasm, optimized the SRAP–PCR reaction system, and obtained and analyzed the whole genome sequence (to be published). On this basis, this study performed transcriptome sequencing on samples from three growth and development stages: primordia (SE8–P), fruiting body differentiation (SE8–F), and mature fruiting body (SE8–M). The transcriptome sequence of the *S. edulis* strain was obtained using Illumina NovaSeq platform technology. By comparing different growth and development stages, DEGs were obtained and enriched for analysis to screen genes and metabolic pathways that may be involved in the growth and development of *S. edulis*. The discovery of these genes and metabolic pathways can not only improve and enrich the transcriptome data of *S. edulis* but also provide some assistance for the study of the growth and development mechanism of *S. edulis* and other edible mushrooms and provide a theoretical basis for genetic breeding research of edible mushrooms.

## 2. Materials and Methods

### 2.1. Strain and Collection of Samples at Different Developmental Stages

**Figure 1:**
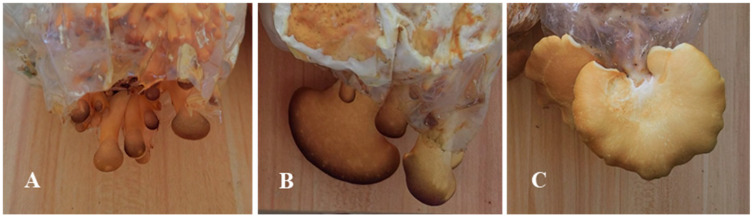
Development stage of *S. edulis* strain SE8. (**A**): Primordia (SE8–P). (**B**) Fruiting body differentiation (SE8–F). (**C**) Mature fruiting body (SE8–M).

The strain used for transcriptome sequencing of *S. edulis* was SE8, characterized by a yellow–brown cap. It was collected from Baishan City, Jilin Province, and preserved at Jilin Agricultural Science and Technology College. The strain was inoculated into a sawdust culture medium (sawdust 78%, wheat bran 20%, lime 1%, gypsum 1%, pH 6.0), with 250 g of dry material per bag, cultivation temperature of 15 °C, humidity of 90%, and natural light. Samples were collected during the three stages of primordia (the 5th day of fruiting), fruiting body differentiation (the 10th day of fruiting), and mature fruiting body (the 15th day of fruiting) (Figure 1), with three biological replicates per stage. The samples were mixed evenly according to the same location and stored in a −80 °C freezer for transcriptome sequencing.

### 2.2. RNA Isolation, Library Construction, and Sequencing

To summarize the gene expression profile of *S. edulis* at three developmental stages, cDNA samples were prepared from the primordia (SE8–P), fruiting body differentiation (SE8–F), and mature fruiting body (SE8–M). The libraries were sequenced on an Illumina NovaSeq platform to generate 150 bp paired-end reads, according to the manufacturer’s instructions. Total RNA was extracted from the sample using a Tiangen DP441 assay kit (Tiangen Biotechnology, Beijing, China). Nanodrop 2000 (Thermo Fisher Scientific, Wilmington, DE, USA) and Agilent 2100 (Agilent Technologies, Santa Clara, CA, USA) were used to detect the concentration and integrity of extracted RNA. Each stage was sampled in triplicate, resulting in a total of 9 libraries. The raw data has been submitted to NCBI with the login number PRJNA1267801.

### 2.3. Analysis of Differentially Expressed Genes (DGEs)

Using Trinity software (2.14.0) [23], clean data were assembled into sequences to obtain the Unigene library of *S. edulis* and obtain transcriptome data. DIAMOND (v2.0.4) [24] software was used to combine the Unigene sequence with Swiss–Prot [25], NR [26], COG [27], KOG, GO [28], eggNOG4.5 [29], and KEGG [30,31] to compare with the database to obtain annotation information for Unigene. Using Bowtie [32], the reads obtained from sequencing were compared with the Unigene library. Based on the comparison results, RSEM (v1.2.19) was used to estimate the expression level, and FPKM values were used to represent the expression abundance of corresponding Unigenes [33]. Differential analysis was performed using DESeq2 software (1.30.1), and the screening criteria for differential genes were FDR < 0.01 and |log2 FC| ≥ 2. Functional annotation of DEGs in the database was performed using the database [34].

### 2.4. Validation of DEGs by qRT–PCR

Primer validation was a good practice for confirming the efficiency of qRT–PCR primers. To ensure the accuracy of RNA–Seq data, six representative DEGs were selected for qRT–PCR. The RNA used for qRT–PCR validation was the same RNA aliquots used for transcriptome sequencing. The FPKM values of these genes were available and showed significant changes in the transcriptional expression data of 9 samples. According to RNA seq data, the expression of the Actin gene was stable at three developmental stages and was therefore used as an internal reference gene (Table S1). Primer v5.0 was also used to design specific primers for internal reference genes and DEGs (Table S2). RNA extraction was performed using the RNAprep Pure polysaccharide polyphenol plant total RNA extraction kit (DP441) manufactured by Tengen (Wenzhou, China), and PrimeScript provided by Takara was used. The RT reagent kit with gDNA Eraser (Perfect Real Time) synthesis kit was used to synthesize cDNA. Specific primer pairs were obtained from transcriptome data and subsequently used for qRT–PCR. qRT–PCR was performed using the C1000 real-time PCR detection system (Bio–Rad, Hercules, CA, USA) in triplicate. The reaction mixture consisted of 10 μL, which included 5 μL of 1 × ChamQ Blue Universal SYBR qPCR Master Mix (Novozymes, Bagsværd, Denmark), 3 μL of diluted cDNA, and 1 μL of specific forward and reverse primers each. The 2 ^−ΔΔCt^ method was used to analyze gene expression data [35].

## 3. Results

### 3.1. Global Transcriptomic Analysis

**Figure 2:**
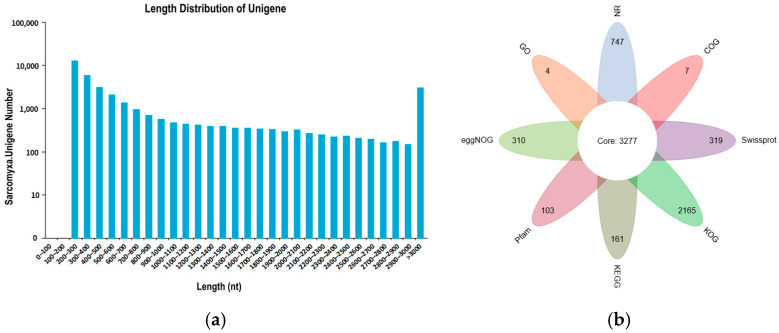
Transcriptomic analysis of SE8. (**a**) Unigene length distribution map. The horizontal axis represents the different length intervals of Unigene; the vertical axis represents the number of Unigenes within a certain length interval. (**b**) Unigene annotation petal diagram.

Transcriptome sequencing was performed on nine samples from three distinct growth and development stages of *S. edulis* strains, yielding a total of 54.67 Gb of clean data. The clean data for each sample reached 5.91 Gb, with a GC content of approximately 51% and a Q30 base percentage of 95.22% or higher. The data indicators performed well, providing a reliable data basis for subsequent analysis (Table S3). Following assembly, a total of 36,423 Unigenes were obtained, with an N50 of 2335. Of these, 8986 Unigenes were longer than 1 kb (Figure 2a). Functional annotation of the Unigene sequences was conducted, resulting in 21,206 annotated Unigenes. Among these, 3277 genes were shared across annotations, with the distribution of genes in each annotation category illustrated in the petals (Table 1, Figure 2b)

### 3.2. Gene Expression Level Analysis

**Figure 3:**
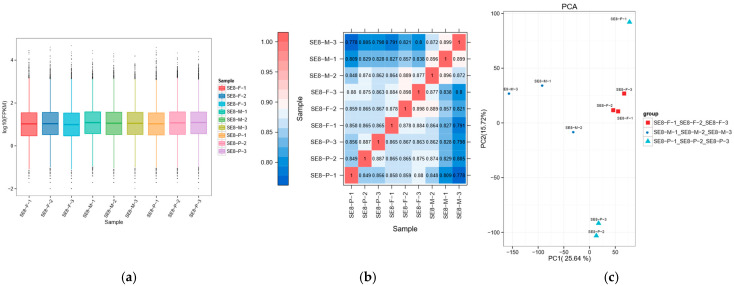
Gene expression level analysis of SE8. (**a**) Box plot of gene expression level distribution (FPKM) in different samples. The horizontal axis in the figure represents different samples; the vertical axis represents the logarithmic value of the sample expression level FPKM. This graph measures the expression levels of each sample from the perspective of the overall dispersion of expression levels. The vertical axis of the point represents the probability density. The peak of the distribution curve represents the region with the highest concentration of gene expression in the entire sample. (**b**) Correlation heatmap of different sample types. The horizontal and vertical axes in the figure are sample numbers, and the colors reflect the correlation between the samples. The corresponding relationship is shown in the legend on the left. (**c**) Principal Component Analysis Diagram. The coordinates PC1 and PC2 in the figure represent the principal components of different samples, and percentages represent the contribution values of corresponding principal components to sample differences. Each point represents a sample, and samples from different groups were represented by different colors and shapes.

The analysis of gene expression levels showed that the density of the nine samples was relatively concentrated, with log10 (FPKM) values ranging from −1.09 to 3.17 (Figure 3a). Principal component analysis and Spearman correlation coefficient rho analysis showed good sample repeatability, significant inter-group differences among samples at different developmental stages, and clear clustering among samples at the same developmental stage. These results demonstrate the stability and reliability of the test data. A stable data foundation for subsequent differential gene screening and enrichment analysis is provided (Figure 3b,c, Table S4).

### 3.3. Identification of DEGs Across Various Developmental Stages

**Figure 4:**
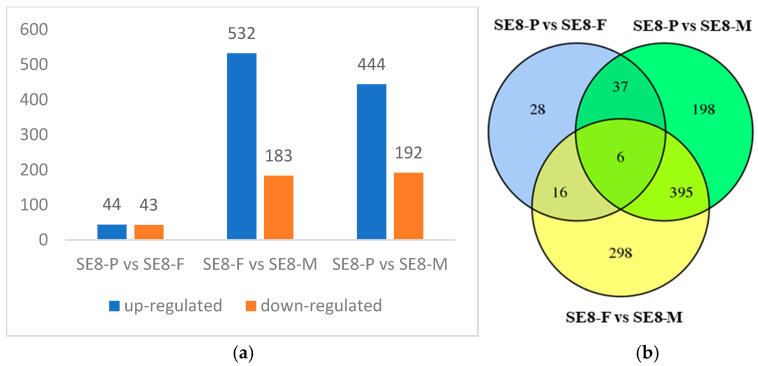
Differentially expressed genes of SE8. (**a**) Statistics of differentially expressed genes. (**b**) Venn diagram of a differentially expressed gene.

This study identified DEGs in the SE8 strain at three stages: primordia (SE8–P), fruiting body differentiation (SE8–F), and mature fruiting body (SE8–M). There were 87 DEGs between SE8–P and SE8–F, with 44 genes upregulated and 43 genes downregulated. There were 715 DEGs between SE8–F and SE8–M, with 532 genes upregulated and 183 genes downregulated. There were 636 DEGs between SE8–P and SE8–M, with 444 genes upregulated and 192 genes downregulated (Figure 4a). To visually demonstrate the overlap of DEGs between different comparison groups, this study conducted a Venn diagram analysis on the DEGs of different comparison combinations. Among them, there were 6 common genes, 28 unique DEGs in SE8–P vs. SE8–F, 298 unique DEGs in SE8–F vs. SE8–M, and 198 unique DEGs in SE8–P vs. SE8–M (Table 2, Figure 4b).

### 3.4. KOG Functional Classification of DEGs

**Figure 5:**
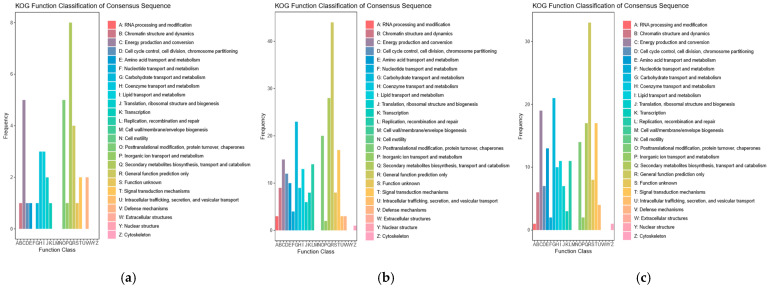
Statistical chart of KOG annotation classification for DEGs. (**a**) SE8–P vs. SE8–F, (**b**) SE8–F vs. SE8–M, (**c**) SE8–P vs. SE8–M.

This study performed KOG functional annotation on DEGs from the SE8 strain at various developmental stages. General function predictions only were made according to KOG annotations, post-translational modification, protein turnover, chaperones, energy production and conversion, coenzyme transport and metabolism, lipid transport and metabolism, secondary metabolite biosynthesis, transport, and catabolism. There was a high enrichment in the three stages of SE8–P vs. SE8–F, SE8–F vs. SE8–M, and SE8–P vs. SE8–M, which were mainly related to the synthesis, transportation, and metabolism of organic matter (Figure 5).

### 3.5. GO Annotation Analysis of DEGs

**Figure 6:**
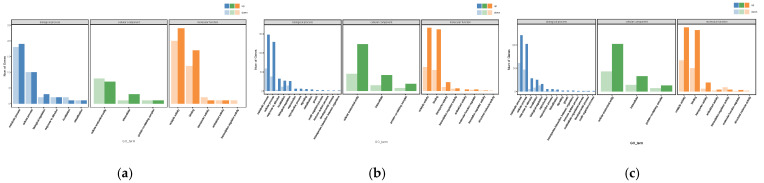
GO annotation of DEGs. (**a**) SE8–P vs. SE8–F, (**b**) SE8–F vs. SE8–M, (**c**) SE8–P vs. SE8–M.

The GO annotation includes three categories: Molecular Function (MF), Cellular Component (CC), and Biological Process (BP). The GO analysis during the growth process of the *S. edulis* strain is shown in Figure 6, and the results indicate that DEGs were involved in various biologically significant processes. In the comparison of SE8–P vs. SE8–F, SE8–F vs. SE8–M, and SE8–P vs. SE8–M, the significantly enriched terms were almost the same. The differential gene numbers enriched in the metabolic process and cellular process in BP, cellular anatomical entity and intracellular in CC, and catalytic activity and binding entries in MF were significantly higher than those in the other entries.

During the differentiation stage of primordia and fruiting bodies (SE8–P vs. SE8–F), 60 DEGs were annotated, of which 29 genes were upregulated and 31 genes were downregulated. These DEGs were distributed among 34 BP, 7 CC, and 54 MF components. The enriched BP terms include Sphingomyelin catabolic process, Polyphosphate metabolic process, plasma membrane selenite transport, Cyanamide metabolic process, Carnitine biosynthetic process, and Nitrile metabolic process. This was related to the decomposition, synthesis, and metabolism of compounds. The enriched CC terms include Integral component of plasma membrane, Integral component of membrane, Host cell nucleus, Nucleus, and Cytoplasm. This was related to the formation of the nucleus, cytoplasm, and membrane. The enriched MF terms include Oxidoreductase activity, Monooxygenase activity, Heme binding, and Iron ion binding, this was related to oxidative stress response and reactant binding.

During the differentiation and maturation stages of the fruiting body (SE8–F vs. SE8–M), 425 DEGs were annotated, with 312 genes upregulated and 113 genes downregulated. These DEGs were distributed among 741 BP, 122 CC, and 260 MF components. The enriched BP terms include Vitamin biosynthetic process, Water-soluble vitamin metabolic process, Regulation of protein serine/threonine kinase activity, and Phospholipid catabolic process. This was related to vitamin biosynthesis and metabolism, as well as amino acid kinase activity. The enriched CC terms include Integral component of membrane, Nucleosome, Host cell nucleus, Extracellular space, and Cyclin-dependent protein kinase holoenzyme complex. This was related to the formation of the cell nucleus, membrane, and protein kinase complexes. Enriched MF is a term that includes Oxidoreductase activity, 5′–flap endonuclease activity, FAD binding, Carboxy–lyase activity, Zinc ion binding, monooxygenase activity, and Iron ion binding. This was related to oxidative stress response and reactant binding.

In total, 372 DEGs were annotated during the maturation stages of primordia and fruiting bodies (SE8–P vs. SE8–M), with 252 genes upregulated and 120 genes downregulated. These DEGs were distributed among 712 BP, 112 CC, and 240 MF components. The enriched BP terms include Vitamin biosynthetic process, Water-soluble vitamin biosynthetic process, Vitamin metabolic process, Xenobiotic metabolic process, Response to xenobiotic stimulus, and Nitrile metabolic process. This was related to compound metabolism, vitamin biosynthesis metabolism, and stress response. The enriched CC terms include Protein kinase complex, Cyclin-dependent protein kinase holoenzyme complex, Serine/threonine protein kinase complex, Host cell nucleus, and Incipient cellular bud site; this was related to protein kinase complexes and cell formation. The enriched MF terms include Cyanamide hydratase activity, Monooxygenase activity, Iron ion binding, Peroxidase activity, Oxidoreductase activity, and Glucan 1,4–alpha–glucosidase activity. This was related to oxidative stress response and reactant binding (Figure 6).

### 3.6. KEGG Annotation Analysis of DEGs

**Figure 7:**
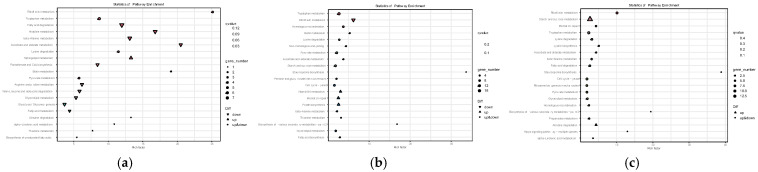
Classification of differentially expressed genes KEGG. (**a**) SE8–P vs. SE8–F, (**b**) SE8–F vs. SE8–M, (**c**) SE8–P vs. SE8–M.

In order to further elucidate the biological pathways associated with DEGs, the top 20 enriched pathways were selected for KEGG enrichment analysis of DEGs in the three stages (Figure 7); 53 DEGs were annotated between SE8–P and SE8–F, and these DEGs were annotated into 29 KEGG entries. The most significantly enriched pathways included Tryptophan metabolism, Histidine metabolism, Lysine degradation, Riboflavin metabolism, Ascorbate and aldarate metabolism, Pyruvate metabolism, Fatty acid degradation, and beta–Alanine metabolism. These enriched pathways indicate that during the process from the primordium to the cap formation stage, the formation of the cap may be promoted by regulating amino acid metabolism activity. A total of 330 DEGs were annotated between SE8–F and SE8–M, and these DEGs were annotated into 88 KEGG entries. The most significantly enriched pathways included Riboflavin metabolism, Tryptophan metabolism, Lysine degradation, Pyruvate metabolism, Pentose and glucuronate interconversions, Starch and sucrose metabolism, Biotin metabolism, and Propanoate metabolism. The enrichment of these pathways suggests that during the formation of fruiting bodies, cells may accumulate substances and promote fruiting body maturation by participating in the synthesis of carbohydrate compounds and various amino acid metabolism activities. A total of 288 DEGs were annotated between SE8–P and SE8–M, and these DEGs were annotated into 87 KEGG entries. The most significantly enriched pathways included Riboflavin metabolism, Lysine degradation, Tryptophan metabolism, Fatty acid degradation, Pyruvate metabolism, beta–Alanine metabolism, Starch and sucrose metabolism, and Pantothenate and CoA biosynthesis. The enrichment of these pathways indicates that during the formation stage of fruiting bodies, substance accumulation and fruiting body maturation can be promoted by coordinating the synthesis and metabolism of various secondary metabolites, carbohydrate compounds, amino acid biosynthesis, and degradation.

This study conducted KEGG enrichment analysis on three stages: primordia (SE8–P vs. SE8–F), mature fruiting body (SE8–F vs. SE8–M), and primordia towards mature fruiting body (SE8–P vs. SE8–M). The statistical results showed that a total of 28 pathways were enriched in all three stages (Table 3). Among them, Tryptophan metabolism (ko00380), Starch and sucrose metabolism (ko00500), Carbon metabolism (ko01200), Riboflavin metabolism (ko00740), Lysine degradation (ko00310), Pyruvate metabolism (ko00620), and Glycerolipid metabolism (ko00561), which were eight pathways related to substance metabolism and degradation, have a relatively large number of enriched genes, with upregulation and downregulation of DEGs in each stage. In Pantothenate and CoA biosynthesis (ko00770), DEGs showed a downregulation trend in all three stages, while in Atrazine degradation (ko00791), DEGs showed an upregulation trend in all three stages. In addition, Basal transcription factors (ko03022), Ubiquitin-mediated proteolysis (ko04120), Ribosome (ko03010), and Phosphatidylinositol signaling system (ko04070) were the four pathways that only appeared in the SE8–F vs. SE8–M stage. Selenocompound metabolism (ko00450) and Mitophagy yeast (ko04139) pathways only appear in the SE8–P vs. SE8–M stage.

### 3.7. Tryptophan Metabolism

**Figure 8:**
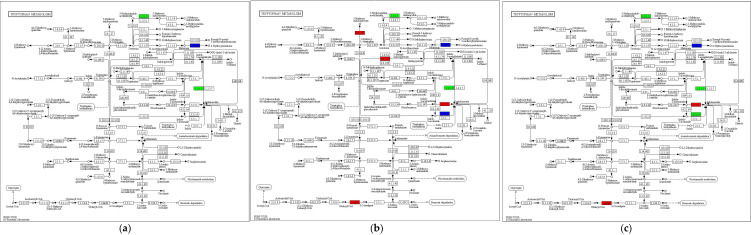
The DEGs in SE8–P vs. SE8–F, SE8–F vs. SE8–M, and SE8–P vs. SE8–M were mapped to Tryptophan metabolism (ko00380). The red represents upregulation, the green represents downregulation, and the purple represents upregulation and downregulation. (**a**) SE8–P vs. SE8–F, (**b**) SE8–F vs. SE8–M, (**c**) SE8–P vs. SE8–M.

KEGG enrichment analysis showed that Tryptophan metabolism (ko00380) contained the highest number of DEGs across the three developmental stages, with more genes downregulated than upregulated during growth and development (Table S5, Figure 8). During primordia-to-fruiting body differentiation (SE8–P vs. SE8–F), a total of seven DEGs were detected, including two upregulated genes, mainly encoding cytochrome P450 monooxygenases, and five downregulated genes, primarily encoding tryptamine 4–monooxygenase, NADPH–cytochrome P450 reductase, and aldehyde dehydrogenases. In the transition from fruiting body differentiation to the mature fruiting body (SE8–F vs. SE8–M), 16 DEGs were identified, with 6 upregulated genes, mainly encoding acetamidase, MFS-type transporter OryC, FAD-linked oxidoreductase, L–tyrosine:2–oxoglutarate aminotransferase (Amt1), and cytochrome P450 monooxygenases, while 10 downregulated genes mainly encoded multifunctional cytochrome P450 monooxygenases, cytochrome P450 monooxygenases, aldehyde dehydrogenases, aspirochlorine biosynthesis protein N, tryptamine 4–monooxygenase, and β–apo–4′–carotenal oxidase. During the primordia-to-mature fruiting body transition (SE8–P vs. SE8–M), 12 DEGs were detected, including 5 upregulated genes, mainly encoding cytochrome P450 monooxygenases, MFS-type transporter OryC, and cyanide hydratase, and seven downregulated genes, primarily encoding cytochrome P450 monooxygenases, bifunctional cytochromes, tryptamine 4–monooxygenase, aldehyde dehydrogenases, and aspirochlorine biosynthesis protein N. According to Table 4 and Figure 8, it can be seen that in Tryptophan metabolism, aldehyde dehydrogenase coordinates with Aldehyde oxidase to convert 5–hydroxyindoleacetate to 5–hydroxyindoleacetate, and it coordinates with indole–3–acetaldehyde oxidase to convert Indole3–acetaldehyde to Indooleacetate. In this study, the gene encoding aldehyde dehydrogenase showed a downregulation trend with the growth and development of the fruiting body of the *S. edulis*, and the two were negatively correlated. It was preliminarily inferred that aldehyde dehydrogenase plays a negative regulatory role in the growth of the *S. edulis*. Related studies have shown that indoacetate was involved in regulating cell division and elongation in different parts of the fruiting body, affecting the length, thickness, and normal expansion and size of the stem cap. Its mechanism of action was similar to that of promoting stem node elongation in plants [36,37,38].

### 3.8. qRT–PCR Validation of DEGs at Different Growth and Development Stages

**Figure 9:**
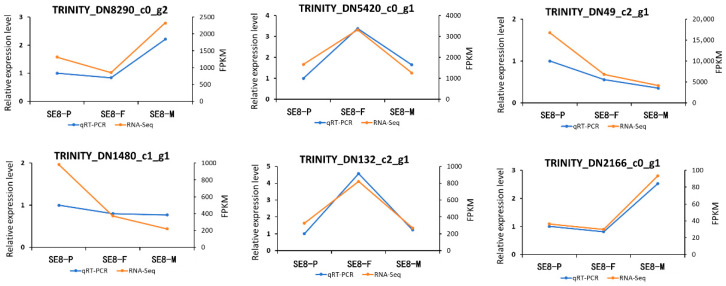
Verifies the expression profile of genes related to the growth and development of *S. edulis* through qRT–PCR analysis.

In order to confirm the transcriptome data, this study screened six highly expressed genes for qRT–PCR to examine the expression patterns of genes related to the growth and development of *S. edulis*. The qRT–PCR results were consistent with the RNA–Seq results, demonstrating the reliability of the RNA–Seq data through the consistency between the two methods (Figure 9). The TRINITYDN1480uc1_g1 gene was a common gene among the three growth and development stages and shows significant expression. According to the annotation library, the gene mainly encodes aldehyde dehydrogenase, which shows a decreasing trend in FPKM value during the three growth and development processes of *S. edulis*.

### 3.9. Genes and Proteins Potentially Critical for Growth and Development of S. edulis

The analysis of DEGs during the growth and development of *S. edulis* (Table 4) showed that, compared with the fruiting body formation stage (SE8–P vs. SE8–F), the gene encoding AB hydrolase superfamily protein was upregulated in the primordium stage, while the gene encoding aldehyde dehydrogenase family was downregulated. Compared with the mature stage of the fruiting body (SE8–F vs. SE8–M), the genes encoding AB hydrolase superfamily protein, aldehyde dehydrogenase family, and Thaumatin family were downregulated during the formation stage of the fruiting body, while the genes encoding glycoside hydrolase family, Aldo/keto reductase family, and Eukaryotic aspartyl protease were upregulated. Upregulation of the Amidohydrolase gene was also noted. In the DEGs between the primordium stage and the fruiting body maturation stage (SE8–P vs. SE8–M), the genes encoding the glycoside hydrolase family were upregulated, while the genes encoding the fungal hydrophobin were downregulated. These proteins can promote the growth and development of *S. edulis* [39].

## 4. Discussion

### 4.1. Comparative Analysis of the Transcriptome of Edible Fungi

Transcriptome sequencing technology can comprehensively and quickly obtain mRNA sequence information transcribed by specific tissues or organs of a species in a certain state and study gene expression levels and structures at the overall level, revealing molecular mechanisms in specific biological processes. Non-parametric transcriptome sequencing technology has the characteristics of high throughput, high resolution, wide applicability, high sensitivity, and quantitative dynamics and was the foundation and starting point for gene function and structure research. In recent years, with the popularization of high-throughput sequencing technology, transcriptome research on edible fungi has shifted from single-species functional annotation to cross-species comparative analysis. At present, relevant research has been conducted on typical edible mushrooms such as *Auricularia auricula*, *Ganoderma lucidum*, *Hericium erinaceus*, *Dictyophora indusiata*, *Lentinula edodes*, *Pleurotus ostreatus*, and *Flammulina velutipes*. The main research focuses on the gene expression differences in the growth and development, environmental adaptation, secondary metabolism, and morphogenesis of edible fungi under different developmental stages, strains, and environmental stresses [40,41]. These findings provide valuable insights into the evolutionary strategies and functional differentiation of edible fungi. Wen [42] conducted multi-omics sequencing on *D. indusiata* balls and mature *D. indusiata*, and the results showed that the key to the morphological development of *D. indusiata* balls after harvesting was stem elongation, with the fastest elongation rate in the middle of the stem. Fu [43] conducted comparative transcriptomic analysis on the mycelium, primordia, and fruiting bodies of *Pleurotus Nebrodensi*, identifying DEGs involved in morphogenesis, primary carbohydrate metabolism, cold stimulation, and blue light response. Tang [44] conducted transcriptomic studies on three treatments of *L. edodes* hyphae (light avoidance culture for 30 days, light avoidance culture for 80 days, and light avoidance for 30 days plus 50 days), revealing the molecular mechanism of light-induced *L. edodes* color transformation. It was found that photosensitive genes, light signal transduction pathways, and pigment-forming genes were involved in *L. edodes* color transformation. Wang [45] identified a total of 8495 Unigenes, 4047 proteins, and 30 metabolites in the de novo sequencing analysis of *Morchella importuna*. These results provide a basis for understanding the taste formation, texture regulation, and color changes in fruiting bodies. Wang [46] subjected the hyphae, primordia, fruiting bodies, and spores of *P. ostreatus* to high-temperature stress, H_2_O_2_ stress, NaCl stress, and pH stress to detect the expression levels of peroxidase (CAT). The results showed that CAT responded most significantly to high-temperature stress; however, the response mechanisms of different CAT to high temperatures were different. Zhang [47] found through transcriptome KEGG enrichment analysis of the growth and development stages of *Hypsizygus marmoreus* fruiting bodies that the color transition stage was associated with MAPK, cAMP, and blue light signaling pathways. Light affects the expression of genes related to the initiation of *H. marmoreus* fruiting bodies, and nitrogen stress may enhance fruiting body maturation. Zhou [48] analyzed the DEGs between the hyphae and fruiting bodies of *Auricularia polytricha* and found that tyrosinase had the highest expression level in the fruiting bodies, which may be an important regulatory gene in pigment synthesis in *A. polytricha* fruiting bodies. Ma [49,50] analyzed the pigment synthesis pathway of *Auricularia cornea* through multiple omics studies and identified the main pigments (γ–glutamyl–3,4–dihydroxybenzoate), major intermediates (α–D–glucose–1P, citrate, 2–oxogluconate, and glutamate), and key enzymes, such as phenoloxidase, in the fruiting body of *A. cornea*. Li [51] used corn cob and tobacco straw as two different substrates to cultivate *P. ostreatus*. Based on this, the DEGs and metabolic pathways of *P. ostreatus* at different developmental stages were analyzed. The results revealed the transcriptome adaptation of *P. ostreatus* to tobacco straw and provided new insights for the molecular mechanism of biomass conversion by edible bacteria using straw. Duan [12] studied *S. edulis* mycelium growing to a half bag (B1), mycelium in cold stimulation after a full bag (B2), mycelium in primordia appearing (B3) and primordia (B4), mycelium at the harvest stage (B5), and mature fruiting body (B6). They performed transcriptome sequencing for the six growth and development stages of *S. edulis* to screen for genes related to lignin degradation. The materials used in this study were *S. edulis* strains collected from the field, and transcriptome sequencing was performed by sampling tissues from three typical stages: primordia (SE8–P), fruiting body differentiation (SE8–F), and mature fruiting body (SE8–M). The results of this study showed significant differences in DEGs among different tissue types, especially in the SE8–F vs. SE8–M stage, where the number of DEGs was significantly higher than in the SE8–P vs. SE8–F stage and SE8–P vs. SE8–M stage. GO and KEGG enrichment analysis of DEGs between different tissue types showed the same functional distribution trend of DEGs (Figure 5, Figure 6 and Figure 7), confirming that mature fruiting bodies were the main gene expression tissues in the formation process. This study preliminarily speculated that the genes encoding aldehyde dehydrogenase and fungal hydrophobin play a negative regulatory role in the growth and development of *S. edulis* through GEG screening and KEGG enrichment analysis between different growth and development stages. Related studies have shown that the aldehyde dehydrogenase gene was an important gene discovered in recent years that can regulate plant branching (tillering). It metabolizes endogenous and exogenous aliphatic and aromatic aldehyde molecules into corresponding carboxylic acids [52,53]. Fungal hydrophobin was crucial for the formation of fungal aerial hyphae, but it has a negative regulatory effect on the development of fruiting bodies [54,55], which was consistent with the results of this study. The genes encoding glycoside hydrolase and AB hydrolase superfamily proteins play a positive regulatory role. Related studies have shown that the GH16 family contains multiple glycoside hydrolases, and its members have activity towards various glycosidic bonds of pectin; these hydrolases play a role in the degradation of lignocellulose [56]. The AB hydrolase superfamily protein assists in disrupting the structure of plant cell walls, creating conditions for glycoside hydrolase to degrade cellulose and hemicellulose, allowing hyphae to absorb and utilize nutrients and promote the growth and development of fruiting bodies. Although both this study and Duan’s [12] used *S. edulis* strains, there were differences in the source of the strains and the transcriptome sequencing techniques used, as well as different research purposes, resulting in certain differences in the research results. This also provides a data reference for the genetic diversity analysis of *S. edulis* and enriches its genetic information.

### 4.2. Signal Pathways Involved in the Development of Fruiting Bodies in S. edulis

The development of fruiting bodies was one of the most critical stages in the life cycle of edible fungi, involving complex metabolic regulatory networks. This process was regulated by multiple metabolic pathways, including various carbohydrate metabolism, signal transduction, synthesis of secondary metabolites, and cell wall remodeling. During the developmental stage of the fruiting body, a large amount of carbon sources and energy was required, among which glycolysis and the tricarboxylic acid cycle were core energy metabolism pathways [57,58]. The genes encoding hexokinase [59], phosphofructokinase (PFK) [60], and trehalose–6–phosphate synthase (TPS) [61] were crucial for hyphal development and fruiting body formation. In addition, the development of fruiting bodies was also regulated by environmental signals (such as light and temperature) and intracellular signaling pathways, including cAMP and MAPK [62]. In *Agaricus bisporus*, MAPK signaling promotes the aggregation of fungal filaments into primordia by inhibiting hyphal branching-related genes. In *A. bisporus*, MAPK signaling promotes the aggregation of fungal filaments into primordia by inhibiting hyphal branching-related genes [63]. During the development of fruiting bodies, a large number of secondary metabolites were synthesized, including terpenes [64,65,66], polyphenols [67], and sterols [68], which can promote cap differentiation and may also affect spore release. The morphological construction of sub-entities relies on the dynamic remodeling of cell wall polysaccharides. Chitin synthase (CHS) and β–β–glucan synthase (FKS) genes promote the formation of the cell wall cytoskeleton [69]. Lignin-degrading enzymes (such as laccase and manganese peroxidase MnP) enhance the mechanical strength of organelles by modifying the cross-linked structure of cell walls [70]. In this study, a total of 28 pathways appeared in all three stages of *S. edulis* growth and development. The pathways with more enriched genes were mostly related to substance metabolism and degradation, including tryptophan metabolism, starch and sucrose metabolism, carbon metabolism, riboflavin metabolism, lysine degradation, pyruvate metabolism, glycerolipid metabolism, and glycolysis/gluconeogenesis. In addition, the Pantothenate and CoA biosynthesis pathways showed a downregulation trend of DEGs in all three growth and development stages, while the Atrazine degradation pathway showed an upregulation trend of DEGs in all three growth and development stages. The discovery of these pathways provides a valuable reference for research on the development of *S. edulis* fruiting bodies. At present, although relevant pathways have been discovered, further analysis of the genes and metabolites related to the pathways has not been carried out. Further in-depth analysis should be conducted in combination with metabolomics and proteomics.

### 4.3. The Role of Tryptophan in Edible Fungi

Tryptophan was a precursor of bioactive substances such as niacin, serotonin, melatonin, etc. It not only participates in the synthesis and metabolic regulation of proteins in the body but also affects the metabolism of nutrients such as proteins, sugars, and fats. It can also participate in the growth and development of plants, thereby affecting the development of edible mushroom fruiting bodies [71,72]. Tryptophan plays multiple roles in the growth and development of edible fungi. It not only participates in protein synthesis as an essential amino acid but also acts as a precursor for secondary metabolites. It affects hyphal growth, fruiting body differentiation, and stress resistance by regulating signaling pathways [73,74]. Yan [75] conducted comparative transcriptome analysis on different forms of *L. edodes* fruiting bodies and found that amino acid metabolism pathways, such as trypsin metabolism, were significantly enriched in malformed *L. edodes* fruiting bodies. Genes encoding heat shock proteins, G proteins, and β –1,3–glucanase in the GH5 family were involved in the development of *L. edodes* fruiting bodies. Duan [76] conducted transcriptome sequencing on the five growth and development stages of *D. indusiata*, and the results showed that the cap was the main source of indole–3–acetic acid synthesis, confirming the importance of tryptophan metabolism in the differentiation of *D. indusiata* fruiting bodies. At the same time, three new genes related to the tryptophan metabolism pathway, IAA synthesis, were identified in the cap. In this study, five genes encoding aldehyde dehydrogenase were screened in Tryptophan metabolism. These genes were negatively correlated with the growth and development of the fruiting body of the *S. edulis* and showed significant expression of aldehyde dehydrogenase in the primary stage, which may promote the formation of hyphal tangles. As the fruiting body matures, the expression level of Aldehyde dehydrogenase was downregulated, suggesting that it may promote stem elongation and cap formation by regulating the formation of indoacetate, leading to a shift in the development of the fruiting body from the cap growth stage to the fruiting body maturity stage of *S. edulis*. Aldehyde dehydrogenase was an important enzyme class in the process of clearing reactive oxygen species in organisms, which can catalyze the oxidation of toxic aldehydes and the rapid dehydrogenation of toxic aldehydes, further enhancing the cell’s aldehyde detoxification defense mechanism [77,78]. It can also regulate the growth and development of microorganisms and has been reported in various microorganisms such as *Escherichia coli* [79], *Saccharomyces cerevisiae* [80], *Tricholoma vaccinum* [81], *P. ostreatus* [82], and *Pleurotus geesteranu* [83]. At present, this study has only preliminarily screened the gene and has not yet verified its function. In the future, the gene can be accurately identified and further analyzed based on metabolomics and proteomics data to elucidate its mechanism of action in the growth and development of *S. edulis*.

## 5. Conclusions

The completion of transcriptome sequencing provides an effective means for studying the genetic mechanism and molecular breeding of *S. edulis*. This study obtained 54.67 GB of clean data and 21,206 Unigene annotation results. Functional annotation and enrichment of DEGs between the three growth and development stages of primordia (SE8–P), fruiting body differentiation (SE8–F), and mature fruiting body (SE8–M) were performed using GO and KEGG. At the same time, highly differentially expressed aldehyde dehydrogenase, fungal hydrophobin, glycoside hydrolase, and AB hydrolase superfamily protein genes were identified. Among them, the gene encoding aldehyde dehydrogenase was annotated into the trypsin metabolism (ko00380) pathway through KEGG, and it was preliminarily speculated that aldehyde dehydrogenase forms *S. edulis* fruiting bodies by regulating indoacetate. The accuracy of RNA–Seq and DEG analysis was validated using quantitative PCR. The above research results provide valuable information on the molecular mechanisms of *S. edulis* fruiting body development. This study aims to provide accurate genetic data on the molecular mechanisms of growth and development and enrich the genetic database of *S. edulis*. Although this study identified differential genes that may be related to the growth and development of *S. edulis*, only qRT–PCR was performed. Further functional validation and cloning of the screened differential genes were needed to clarify their mechanisms of action in growth and development. At the same time, this study used non-parametric transcriptome sequencing, which has the characteristics of wide applicability, high sensitivity, and quantitative dynamics. However, compared with parametric transcriptome sequencing, its accuracy was poorer. Future studies will integrate and analyze transcriptome data based on whole-genome data to improve the accuracy of the results.

## Figures and Tables

**Table 1:** Unigene annotation statistics table.

Annotation Database	Annotated Number	300 ≤ Length < 1000	Length ≥ 1000
COG annotation	7457	2791	3156
GO annotation	15,203	5495	6029
KEGG annotation	10,853	3595	4831
KOG annotation	7728	2435	3854
Pfam annotation	12,045	4091	5879
Swissprot annotation	7717	2327	4167
TrEMBL annotation	18,204	6402	7696
eggNOG annotation	12,883	4622	5472
NR annotation	18,261	6421	7712
All annotated Unigenes	21,206	7960	7837

**Table 2:** Information on differentially expressed genes in three stages.

Gene ID	Swissprot Annotation	Nr Annotation
TRINITY_DN327_c0_g1	6,7–dimethyl–8–ribityllumazine synthase	hypothetical protein
TRINITY_DN1035_c0_g1	Nitronate monooxygenase	2–nitropropane dioxygenase
TRINITY_DN3926_c0_g1	-	hypothetical protein
TRINITY_DN5751_c0_g1	Trimethyllysine dioxygenase	hypothetical protein
TRINITY_DN579_c0_g1	3,4–dihydroxy–2–butanone 4–phosphate synthase	3,4–dihydroxy–2–butanone 4–phosphate synthase–domain–containing protein
TRINITY_DN1480_c1_g1	Aldehyde dehydrogenase	aldehyde dehydrogenase

**Table 3:** Statistical analysis of common pathways in the three developmental stages of SE8.

ko ID	Pathway	SE8–P vs. SE8–F			SE8–F vs. SE8–M			SE8–P vs. SE8–M		
		Total	Up	Down	Total	Up	Down	Total	Up	Down
ko00010	Glycolysis/Gluconeogenesis	3	0	3	6	3	3	7	3	4
ko00053	Ascorbate and aldarate metabolism	3	0	3	4	1	3	4	1	3
ko00061	Fatty acid biosynthesis	1	0	1	4	2	2	2	0	2
ko00071	Fatty acid degradation	5	0	5	5	2	3	7	1	6
ko00280	Valine, leucine, and isoleucine degradation	3	0	3	5	1	4	5	1	4
ko00310	Lysine degradation	4	1	3	7	3	4	7	3	4
ko00330	Arginine and proline metabolism	3	0	3	5	1	4	4	0	4
ko00340	Histidine metabolism	4	0	4	4	1	3	3	0	3
ko00380	Tryptophan metabolism	7	2	5	16	6	10	12	5	7
ko00410	beta–Alanine metabolism	4	0	4	5	1	4	6	1	5
ko00500	Starch and sucrose metabolism	1	1	0	11	10	1	13	13	0
ko00511	Other glycan degradation	1	1	0	2	0	2	1	1	0
ko00561	Glycerolipid metabolism	3	0	3	8	5	3	7	3	4
ko00592	alpha–Linolenic acid metabolism	1	0	1	1	1	0	2	1	1
ko00620	Pyruvate metabolism	4	1	3	11	5	6	8	3	5
ko00640	Propanoate metabolism	1	0	1	5	4	1	5	2	3
ko00730	Thiamine metabolism	1	1	0	3	2	1	1	0	1
ko00740	Riboflavin metabolism	4	1	3	7	0	7	10	1	9
ko00770	Pantothenate and CoA biosynthesis	3	0	3	3	0	3	4	0	4
ko00780	Biotin metabolism	2	1	1	4	2	2	1	0	1
ko00791	Atrazine degradation	1	1	0	1	1	0	2	2	0
ko00910	Nitrogen metabolism	1	0	1	3	1	2	2	1	1
ko01040	Biosynthesis of unsaturated fatty acids	1	0	1	1	1	0	2	0	2
ko01200	Carbon metabolism	2	1	1	10	6	4	9	3	6
ko01212	Fatty acid metabolism	2	0	2	5	3	2	4	0	4
ko02010	ABC transporters	1	0	1	2	1	1	1	0	1
ko03440	Homologous recombination	1	0	1	9	6	3	5	3	2
ko04146	Peroxisome	1	0	1	3	2	1	4	2	2

**Table 4:** Genes and proteins are potentially critical for the growth and development of *S. edulis*.

Gene ID	FDR	log_2_ FC	Regulated	Pfam Annotation	Swiss–Prot Annotation
SE8–P vs. SE8–F					
TRINITY_DN281_c2_g1	0.0091	1.1308	up	alpha/beta hydrolase fold	AB hydrolase superfamily protein
TRINITY_DN5420_c0_g1	0.0043	1.0802	up	alpha/beta hydrolase fold	AB hydrolase superfamily protein
TRINITY_DN1480_c1_g1	0.0009	−1.2947	down	Aldehyde dehydrogenase family	Aldehyde dehydrogenase
TRINITY_DN17839_c0_g1	0.0000	−2.1396	down	Aldehyde dehydrogenase family	Aldehyde dehydrogenase
TRINITY_DN32179_c0_g1	0.0001	−2.1490	down	Aldehyde dehydrogenase family	Aldehyde dehydrogenase
TRINITY_DN327_c0_g1	0.0000	−1.1550	down	6,7–dimethyl–8–ribityllumazine synthase	6,7–dimethyl–8–ribityllumazine synthase
SE8–F vs. SE8–M					
TRINITY_DN281_c2_g1	0.0000	−1.4898	down	alpha/beta hydrolase fold	AB hydrolase superfamily protein
TRINITY_DN1890_c0_g1	0.0004	−1.0374	down	alpha/beta hydrolase fold	-
TRINITY_DN4014_c0_g1	0.0001	−1.0902	down	Aldehyde dehydrogenase family	Aldehyde dehydrogenase 5, mitochondrial
TRINITY_DN679_c0_g1	0.0010	−1.2214	down	Aldehyde dehydrogenase family	Beta–apo–4′–carotenal oxygenase
TRINITY_DN1480_c1_g1	0.0059	−1.0289	down	Aldehyde dehydrogenase family	Aldehyde dehydrogenase
TRINITY_DN3228_c0_g1	0.0000	1.1984	up	Glycosyl hydrolases family 16	Short–chain dehydrogenase/reductase VdtF
TRINITY_DN2724_c0_g1	0.0001	1.1198	up	Glycosyl hydrolases family 5	Glucan endo–1,6–beta–glucosidase B
TRINITY_DN6981_c0_g1	0.0053	1.5132	up	Glycosyl hydrolase family 71	Glucan endo–1,3–alpha–glucosidase agn1
TRINITY_DN5133_c0_g1	0.0000	1.7507	up	Glycosyl Hydrolase Family 88	-
TRINITY_DN6759_c0_g1	0.0004	1.5236	up	Glycosyl hydrolase family 76	-
TRINITY_DN9190_c0_g1	0.0000	−1.0044	down	Thaumatin family	-
TRINITY_DN1190_c2_g1	0.0000	−1.1813	down	Thaumatin family	-
TRINITY_DN711_c10_g1	0.0007	1.0551	up	Eukaryotic aspartyl protease	Aspartic protease
TRINITY_DN1448_c0_g1	0.0059	2.2227	up	Eukaryotic aspartyl protease	Aspartic protease
TRINITY_DN1508_c1_g1	0.0017	1.2047	up	Aldo/keto reductase family	Alcohol oxidase
TRINITY_DN8842_c0_g1	0.0044	1.3520	up	Aldo/keto reductase family	NADPH–dependent conjugated polyketone reductase
SE8–P vs. SE8–M					
TRINITY_DN2724_c0_g1	0.0000	1.2785	up	Glycosyl hydrolases family 5	Glucan endo–1,6–beta–glucosidase B
TRINITY_DN6759_c0_g1	0.0009	1.4974	up	Glycosyl hydrolases family 76	-
TRINITY_DN49_c2_g1	0.0000	−2.2034	down	Fungal hydrophobin	Hydrophobin–B
TRINITY_DN270_c0_g1	0.0014	−1.3033	down	Fungal hydrophobin	Lipase 1

## Data Availability

The transcriptome data presented in this study have been deposited in the National Center for Biotechnology Information Sequence Read Archive under accession number PRJNA1267801: https://dataview.ncbi.nlm.nih.gov/object/PRJNA1267801?reviewer=mjt1amaerauoilo1h6f60ic9or (accessed on 27 May 2025).

## Supplementary Materials

The following supporting information can be downloaded at https://www.mdpi.com/article/10.3390/jof11100750/s1. Table S1: Internal reference gene sequence information; Table S2: Primer sequences for internal reference genes and DEGs genes; Table S3: Statistical able of sequencing data evaluation of three strains at different development stages; Table S4: Comparison of correlation between transcriptome samples; Table S5: DEGs encoding annotation results of Tryptophan metabolism (ko00380) at three growth and development stages.

## Abbreviations

COG	Cluster of Orthologous Groups of proteins
GO	Gene Ontology
KEGG	Kyoto Encyclopedia of Genes and Genomes
KOG	Clusters of orthologous groups for eukaryotic complete genomes
Pfam	Protein Families Database
TrEMBL	Translation of EMBL nucleotide sequence database
eggNOG	evolutionary genealogy of genes: Non-supervised Orthologous Groups
NR	Non-Redundant Protein Database
DEG	Differentially expressed gene
SE8–P	Primordia
SE8–F	Fruiting body differentiation
SE8–M	Mature fruiting body
MF	Molecular Function
CC	Cellular Component
BP	Biological Process
*A. bisporus*	*Agaricus bisporus*
*C. cinereus*	*Coprinus cinereus*
*S. edulis*	*Sarcomyxa edulis*
*S. commune*	*Schizophyllum commune*
*P. ostreatus*	*Pleurotus ostreatus*
*D. indusiata*	*Dictyophora indusiata*
*L. edodes*	*Lentinula edodes*
*M. importuna*	*Morchella importuna*
*H. marmoreus*	*Hypsizygus marmoreus*
*A. polytricha*	*Auricularia polytricha*
