# Supplementary material for: Transcriptome Analysis and Its Application in Screening Genes Related to the Growth and Development of Sarcomyxa edulis
Source: J Fungi (Basel). 2025 Oct 20;11(10):750. doi: 10.3390/jof11100750 (PMC12565735; doi:10.3390/jof11100750)
Supplement: Supplementary file 1 [file jof-11-00750-s001.zip › jof-3854706-supplementary.pdf]

# Transcriptome Analysis and Its Application in Screening Genes Related to the Growth and Development of *Sarcomyxa edulis*

**Wanzhu Jiang [1,†], Xiao Huang [2,†], Peng Wang [2], Bilal Ahmad [3], Ting Yang [1], Ziyuan Wang [1], Tianyu Ren [1] and Jize Xu [1,*]**

**Table S1 Internal reference gene sequence information**

>Gene.45191::TRINITY_DN2025_c0_g1::g.45191::m.45191

Gene.45191::TRINITY_DN2025_c0_g1::g.45191 ORF type:complete len:339

(+),score=49.23,sp|Q9Y707|ACT2_SUIBO|60.76|1e-148,Actin|PF00022.20|3e-104

TRINITY_DN2025_c0_g1:350-1366(+)

ATGATTGGTCGTCCCCGTGGGCATGCCTATAGTATGAATCCGGAGGCTTATGTTGGA
AATGAAGCCCAATCAAAGCGCGAAATTCTCGAAATAAATTACCCTATCGAACGTGG
CATCGTGACACACTGGGACGACATGGAGAAAATTTGGCATCACACTTTCCGAGAA
GAGCTCAGAATCATTCCAGACGAGCACCCCATGCTCATAGCTGATACACCTCTGAA
CCCCAAAGCTAACCGCGAGATAACAGCCGAGAAAATGTTCGAGACTTTCAATGCA
CCCGCGATCTACATGTGTATGCAGGCCGTTCTTCCCATGTACGATTGCCATACGACC
GGTATCGTTCTTGATTCGGGCGATGGTGTCACTCACGCTGTGCCCATCTACGAAGG
CTCCCCAGTGCACCATGCTATCGCCCGTCTGGACTTGGCTGGACGCGATTTGACTC
AAGGTCTCACGGATTGCCTCGTCCACCCCACAGAAAAGAGCCAGGATTATCCCGT
CCCCTCCGAGGTTGATGTTATGGGTATCAAAGAGAGTCTCGGCTACGTTGCCCTTG
AATTCGAACAAGAACTCCGGTCTTCAGACATTGAGAAGTCGTACGAATTGCCTGAT
GGCGAAGTCATTGGCGTTGGTAGCGAGCGATTCCGTATCCCAGAAGCGATGTTCCA
ACCTTCCATCCTCGGCCGGGAAGGTCCCGGCATTCACCAGATTATCTACGATTCTAT
TTCAAAATGTGATCCTGAGCTGCACCGTGATCTCTACAGTCGTATCATTCTTTGCGG
AGGAACCACCATGCTTTCTGGTATCGTCGATCGCTTACAGAAGGAAATCACTGCCT
TAGCACCATCTGACATGGAGGTGAAAATCATTGCTCGTCCGGAGCGTAAATACGAC
GCTTGGATTGGTGGATCTATCTTAGCCTCTCAAACTACGGTTAAAAACCTATGGTGC
ACTAAACAAGAGTACGACGAGTTTGGCCCTGACCTCATCCACCGCAAGAACGGTA
ACACTTAG

**Table S2 Primer sequences for internal reference genes and DEGs genes**

| Primers | Primer sequence (5 '-3') |
|---|---|
| DN132-F | ATGGGGTCTATCGGCAAAGC |
| DN132-R | ATCAAGTGGTCGGCAGTATCGT |
| DN1480-F | GATGTTTAGGCGTTTGTGGGT |
| DN1480-R | TCGTGTCGTAAGGATGCTGTTT |
| DN2166-F | GCAGCCGCATTCTTCATCTT |
| DN2166-R | GCGTTTCCCTTTGCTCTCAC |
| DN49-F | CCCTCAGTCACTCACCAAACAG |
| DN49-R | TGGGACTTCTTCCTGGATTCG |
| DN5420-F | TGATTTGCCCGATGTTAGATGA |
| DN5420-R | CTCACCTCATCAGTCCCACGA |
| DN8290-F | GCGACAGGCGACTCAAATAGC |
| DN8290-R | AGCGTGTCACCATTCAACCAA |
| p.Act-F | CTGATACACCTCTGAACCCCAA |
| p.Act-R | CAGCGTGAGTGACACCATCG |

**Table S3 Statistical able of sequencing data evaluation of three strains at different development stages**

| MK-ID | Read Number | Base Number | GC Content | %≥Q30 |
|---|---|---|---|---|
| SE8-P1 | 21,277,228 | 6,321,676,674 | 51.13% | 95.22% |
| SE8-P2 | 20,546,806 | 6,106,827,363 | 51.21% | 95.72% |
| SE8-P3 | 19,889,640 | 5,905,526,347 | 51.15% | 95.67% |
| SE8-F1 | 21,083,584 | 6,266,412,159 | 51.16% | 95.67% |
| SE8-F2 | 20,297,269 | 6,035,654,431 | 51.22% | 95.49% |
| SE8-F3 | 20,204,563 | 5,992,807,093 | 51.30% | 95.71% |
| SE8-M1 | 20,388,116 | 6,053,471,286 | 51.15% | 95.52% |
| SE8-M2 | 20,521,210 | 6,083,221,867 | 51.18% | 95.50% |
| SE8-M3 | 20,107,701 | 5,909,158,320 | 51.30% | 95.75% |

**Table S4 Comparison of correlation between transcriptome samples**

| sample | SE8-P1 | SE8-P2 | SE8-P3 | SE8-F1 | SE8-F2 | SE8-F3 | SE8-M1 | SE8-M2 | SE8-M3 |
|---|---|---|---|---|---|---|---|---|---|
| SE8-P1 | 1 | 0.8499 | 0.8535 | 0.8526 | 0.8556 | 0.8777 | 0.8037 | 0.8444 | 0.7757 |
| SE8-P2 | 0.8499 | 1 | 0.8891 | 0.8604 | 0.8683 | 0.87 | 0.8289 | 0.8731 | 0.804 |
| SE8-P3 | 0.8535 | 0.8891 | 1 | 0.8618 | 0.8637 | 0.8598 | 0.8218 | 0.8597 | 0.7958 |
| SE8-F1 | 0.8526 | 0.8604 | 0.8618 | 1 | 0.8761 | 0.8768 | 0.8228 | 0.8605 | 0.7872 |
| SE8-F2 | 0.8556 | 0.8683 | 0.8637 | 0.8761 | 1 | 0.8961 | 0.8576 | 0.8899 | 0.8227 |
| SE8-F3 | 0.8777 | 0.87 | 0.8598 | 0.8768 | 0.8961 | 1 | 0.8343 | 0.8756 | 0.7997 |
| SE8-M1 | 0.8037 | 0.8289 | 0.8218 | 0.8228 | 0.8576 | 0.8343 | 1 | 0.8947 | 0.8987 |
| SE8-M2 | 0.8444 | 0.8731 | 0.8597 | 0.8605 | 0.8899 | 0.8756 | 0.8947 | 1 | 0.869 |

| | | | | | | | | | |
|---|---|---|---|---|---|---|---|---|---|
| SE8-M3 | 0.7757 | 0.804 | 0.7958 | 0.7872 | 0.8227 | 0.7997 | 0.8987 | 0.869 | 1 |

**Table S5 DEGs encoding annotation results of Tryptophan metabolism (ko00380) at three growth and development stages.**

| Swissprot annotation | SE8-P vs. SE8-F | | SE8-F vs. SE8-M | | SE8-P vs. SE8-M | | Total |
|---|---|---|---|---|---|---|---|
| | up | down | up | down | up | down | |
| Cytochrome P450 monooxygenase | 2 | | 2 | 2 | 3 | 1 | 10 |
| Bifunctional cytochrome P450/NADPH-P450 reductase | | 1 | | | | 1 | 2 |
| Tryptamine 4-monooxygenase | | 1 | | 1 | | 1 | 3 |
| Aldehyde dehydrogenase | | 3 | | 2 | | 2 | 7 |
| Acetamidase | | | 1 | | | | 1 |
| MFS-type transporter oryC | | | 1 | | 1 | | 2 |
| FAD-linked oxidoreductase | | | 1 | | | | 1 |
| L-tyrosine:2-oxoglutarate aminotransferase amt1 | | | 1 | | | | 1 |
| Aspirochlorine biosynthesis protein N | | | | 1 | | 1 | 2 |
| Multifunctional cytochrome P450 monooxygenase | | | | 1 | | | 1 |
| Beta-apo-4'-carotenal oxygenase | | | | 1 | | | 1 |
| Cyanide hydratase | | | | | 1 | | 1 |
| Not annotated | | | | 2 | | 1 | 3 |
| Total | 2 | 5 | 6 | 10 | 5 | 7 | 35 |
